# Public health risk associated with the co-occurrence of aflatoxin B_1_ and ochratoxin A in spices, herbs, and nuts in Lebanon

**DOI:** 10.3389/fpubh.2022.1072727

**Published:** 2023-01-09

**Authors:** Rouaa Daou, Maha Hoteit, Khlood Bookari, Karine Joubrane, Lydia Rabbaa Khabbaz, Ali Ismail, Richard G. Maroun, André el Khoury

**Affiliations:** ^1^Centre d'Analyses et de Recherche (CAR), Unité de Recherche Technologies et Valorisation Agro-Alimentaire (UR-TVA), Faculty of Sciences, Saint-Joseph University of Beirut, Campus of Sciences and Technologies, Mar Roukos, Lebanon; ^2^Faculty of Public Health, Lebanese University, Beirut, Lebanon; ^3^PHENOL Research Group (Public Health Nutrition Program-Lebanon), Faculty of Public Health, Lebanese University, Beirut, Lebanon; ^4^Lebanese University Nutrition Surveillance Center (LUNSC), Lebanese Food Drugs and Chemical Administrations, Lebanese University, Beirut, Lebanon; ^5^University Medical Center, Lebanese University, Beirut, Lebanon; ^6^Department of Clinical Nutrition, Faculty of Applied Medical Sciences, Taibah University, Madinah, Saudi Arabia; ^7^National Nutrition Committee, Saudi Food and Drug Authority, Riyadh, Saudi Arabia; ^8^Department of Food Science and Technology, Faculty of Agricultural Sciences, Lebanese University, Beirut, Lebanon; ^9^Feinberg School of Medicine, Northwestern University, Chicago, IL, United States; ^10^Laboratoire de pharmacologie, Pharmacie clinique et contrôle de qualité des médicaments, Faculty of Pharmacy, Saint-Joseph University of Beirut, Beirut, Lebanon

**Keywords:** contamination, daily exposure, cancer risk, AFB_1_, OTA

## Abstract

**Background:**

Aflatoxin B_1_ and ochratoxin A are mycotoxins produced by filamentous fungi that attack crops on field and storage. Both mycotoxins present a risk on public health since aflatoxin B_1_ is a hepatotoxic and hepatocarcinogenic agent while ochratoxin A can be nephrotoxic. Those mycotoxins can be found in several food items including spices, herbs, and nuts.

**Objectives:**

In Lebanon, few studies address aflatoxin B_1_ and ochratoxin A contamination in spices, herbs, and nuts. So, the aim of this study is to investigate the concentrations of those two mycotoxins particularly in spices and herbs and the concentration of aflatoxin B_1_ in nuts, and to determine the dietary exposure of the Lebanese population and their possible attribution to liver cancer and renal damage.

**Methods:**

In this work, a total of 198 samples of spices, herbs, and nuts were collected from different sites. Aflatoxin B_1_ and ochratoxin A were quantified using immune-affinity columns. A food frequency questionnaire was used to quantify the consumption of spices, herbs, and nuts in Lebanon. Exposure to aflatoxin B_1_ and ochratoxin A was calculated accordingly and liver and kidney cancer risks were evaluated.

**Results:**

Aflatoxin B_1_ was respectively found in 100, 20.4, and 98.6% of the spices, herbs, and nuts samples, while ochratoxin A was found in 100 and 44.4% of spices and herbs, respectively. Aflatoxin B_1_ was found at mean concentration of 0.97, 0.27, and 0.40 μg/kg in spices, herbs, and nuts, respectively while ochratoxin A was found at mean concentrations of 38.8 and 1.81 μg/kg in spices and herbs, respectively. Aflatoxin B_1_ occurrence was shown to be associated in this study with 0.017 additional cancer cases per 100,000 persons per year, and ochratoxin A weekly exposure was shown to be 5.04 ng/kg bw less than the Provisional Tolerable Weekly Intake of 100 ng/kg bw which indicates low risk of renal damage from spices and herbs consumption.

**Conclusion:**

The consumption of spices, herbs, and nuts in Lebanon could lead to an increase in health risks associated with aflatoxin B_1_ and ochratoxin A, specifically spices. The reported occurrence may be directly related to poor storage conditions.

## 1. Introduction

Mycotoxins are secondary derivatives of filamentous fungi produced on-field or during storage due to climatic conditions such as humidity and temperature. Aflatoxins and ochratoxin A (OTA) are types of mycotoxins that can contaminate several foods such as cereals, legumes, nuts, spices, herbs, coffee, cocoa beans, beer, etc. (1–3). Aflatoxins are produced by *Aspergillus* species mainly *Aspergillus flavus* and *Aspergillus parasiticus* (4, 5) while ochratoxin A is produced by *Aspergillus ochraceus, Aspergillus carbonarius, Aspergillus niger*, and *Penicillium verrucosum* (6).

Aflatoxin B1 (AFB_1_) is the most potent of all mycotoxins. It has been classified by the International Agency for Research on Cancer (IARC) as carcinogenic to human (group 1) (7) as it affects the liver and can be the main cause for cases of “acute toxicity, chronic toxicity, carcinogenicity, teratogenicity, genotoxicity, and immunotoxicity.” As for OTA it was classified by IARC as a possible carcinogen to humans (group 2B) (7). Though it is less toxic than AFB_1_, however, exposure to OTA can cause several health consequences like nephrotoxic, hepatotoxic, neurotoxic, teratogenic, and immunotoxic effects (8, 9). OTA was also suspected to be implicated in the Balkan Endemic Nephropathy (BEN) that was characterized by a high prevalence of a unique chronic renal disease in the Balkan region (10–13).

Aflatoxins and OTA are the main mycotoxin contaminants of spices and herbs, while nuts are usually more prone to contamination with aflatoxins. The “Rapid Alert System for Food and Feed” (RASFF) 2018 annual report showed that aflatoxin notifications were mostly reported in nuts while in spices and herbs the main contaminant was OTA (14).

In particular, spices, herbs, and nuts are produced in tropical regions where the climate is characterized by high temperatures, humidity, and rainfalls (15). This climate increases the susceptibility of fungal invasion and mycotoxin production in crops, therefore, increasing the risk of AFB_1_ and OTA contamination. Furthermore, improper harvest, drying, transport, and storage practices can result in further contamination (16).

In Lebanon, spices, herbs, and nuts are very popular and are used extensively in culinary activities so they are frequently imported in large quantities to accomplish the market need (17). Mainly those are imported in batches to get processed and packaged in factories before market distribution or to be handled in bulk for local shops and roasteries. Before admission to the country, the imported products get inspected and tested by the inspectors of the Ministry of Agriculture (MOA) for aflatoxins and OTA contamination (18, 19). Unlike spices, herbs can be produced locally and they are particularly popular due to their wide usage in Lebanese cuisine and due to their medicinal properties (20). So, based on traditional practices, herbs are used in Lebanon in many regions especially rural ones, as therapeutic agents for several health problems (21).

Nonetheless, the weather in Lebanon is characterized by high temperature and humidity (22) so it can induce mycotoxin contamination in spices and herbs in the prevalence of improper agricultural, harvest, drying, and storage practices.

Worldwide, regulations were set to minimize the introduction of contaminated spices to any market. The European Commission (EC) has set a maximum tolerable limit (MTL) of AFB_1_ and OTA in spices at 5 and 15 μg/kg, respectively (23). As for herbs, no MTL is defined by the EC. On the other hand, AFB_1_ MTL in nuts intended for direct human consumption is set at 2 μg/kg for peanuts and other tree nuts, and at 8 μg/kg for almonds and pistachios (23). In Lebanon, “The Lebanese Standards Institution—LIBNOR” follows the European regulations and sets the same MTL for AFB_1_ and OTA in some types of spices and nuts accordingly (19, 24).

On the national level, limited studies have shown the contamination of spices, herbs, and nuts and recommended continuous monitoring of aflatoxins and OTA in the Lebanese market (25–27). A recent study done on spices in Lebanese markets also showed the prevalence of fungal species mostly *A. flavus* even in packaged samples which increases the risk of contamination with AFB_1_ (20). In addition, the RASFF portal reported that several rejection notifications have been issued in multiple European countries to imported batches of spices and nuts from Lebanon due to contamination with AFB_1_ (28).

Therefore, assessing the risk of AFB_1_ and OTA in Lebanon is particularly important especially, since hepatic cancer and kidney disease cases have been increasing (29–31). Accordingly, the aim of this study is to investigate the concentrations of AFB_1_ and OTA particularly in spices and herbs and the concentration of AFB_1_ in nuts, and to determine the dietary exposure of the Lebanese population to those toxins and their possible attribution to liver cancer and renal damage.

## 2. Materials and methods

### 2.1. Sampling

#### 2.1.1. Spices

A total of 73 spices samples were collected from supermarkets and shops in all Lebanese regions including sweet pepper (thirteen), white pepper (ten), black pepper (nine), cinnamon (eight), nutmeg (six), turmeric (three), cumin (three), paprika (four), and spices mix (seventeen). Among the samples collected 51 were vacuum packaged while 22 were purchased unpackaged from traditional local shops.

#### 2.1.2. Herbs

A total of 54 herbs samples were collected from supermarkets and shops in all Lebanese regions including sumac (ten), thyme (ten), coriander (eight), dried mint (six), oregano (six), basil (three), anise (eight), and infused herbs (three). Among the samples collected 34 were vacuum packaged while 20 were purchased unpackaged from traditional local shops.

#### 2.1.3. Nuts

A total of 71 nuts samples were collected from supermarkets and shops in all Lebanese regions including mixed nuts (nine), peanuts (twenty), pistachios (fifteen), cashews (six), almonds (eleven), chickpeas (eight), and others (two). Among the samples collected 28 were vacuum packaged while 43 were purchased unpackaged from traditional local shops.

### 2.2. Sampling area, period, size, and technique

The samples used in the study were collected during the month of January 2020. Samples withdrawn from bulk were collected from all Lebanese governorates, while packaged samples were purchased from different brands representative of the Lebanese market.

Samples were collected according to two different methods. First, if the sample was collected from bulk, the items were mixed thoroughly and 100 g were randomly obtained, however in case the sample was packaged, one package was purchased from every sample. The samples were transferred to the lab afterwards and stored at −18°C until the time of analysis.

### 2.3. Chemicals

Standards of AFB_1_ (2 μg/ml in acetonitrile) were purchased from Sigma-Aldrich (Steinheim, Germany), standards of OTA (50 μg/ml in benzene: acetic Acid 99:1) were purchased from Supelco (Pennsylvania, USA), acetonitrile, methanol, and water (HPLC grade) were purchased from Sigma-Aldrich (Steinheim, Germany), and Aflaochra prep immunoaffinity columns (IAC) specific to aflatoxins and OTA were purchased from R-Biopharm Rhone Ltd. (Glasgow, Scotland, UK). All other chemicals and reagents were of analytical grade.

### 2.4. Sample preparation

#### 2.4.1. Primary preparation

Samples were prepared according to the following method. In case the sample was ground it was mixed thoroughly and then 25 g of the sample were randomly withdrawn to be analyzed. In case the sample was solid, first the sample was grinded and mixed thoroughly, then 25 g were randomly withdrawn to be used as the test sample for analysis.

#### 2.4.2. AFB_1_ and OTA extraction and purification in spices and herbs samples

The extraction of AFB_1_ and OTA was carried out according to the method specified in the Aflaochraprep immunoaffinity columns manual supplied by R-Biopharm Rhone Ltd. (32). First, 25 g of the ground sample were blended using a high speed blender with 5 g of sodium chloride and 100 ml of 80% methanol at a high speed for 2 min. Then the samples were centrifuged at 4,000 rpm for 10 min. Next, 2 ml of the filtrate were diluted with 18 ml of 10% Tween 20 in PBS and filtered through glass microfiber filter paper. After that, 10 ml of the filtrate were passed through the IAC at a slow steady flow rate (2 ml per minute) to purify the toxins. The IAC were then washed with 20 ml PBS to get rid of any sample residues. Finally the toxins were eluted by passing 1 ml of methanol into the IAC followed by 1 ml of distilled water to attain a final volume of 2 ml. The final eluted volumes were collected and stored in sealed vials at proper temperatures until the time of HPLC analysis.

#### 2.4.3. AFB_1_ extraction and purification in nuts samples

The extraction of AFB_1_ and OTA was carried out according to the method specified in the Aflaprep immunoaffinity columns manual supplied by R-Biopharm Rhone Ltd. (33). First, 50 g of the ground sample were blended using a high speed blender with 5 g of sodium chloride and 100 ml of water at a high speed for 2 min. Then 150 ml of methanol were added and the mixture was blended for 2 min. Then the samples were centrifuged at 4,000 rpm for 10 min. Next, 5 ml of the filtrate were diluted with 5 ml of PBS and passed through the IAC at a slow steady flow rate (2 ml per minute) to purify the toxins. The IAC were then washed with 20 ml PBS to get rid of any sample residues. Finally, the toxins were eluted by passing 1 ml of methanol into the IAC followed by 1 ml of distilled water to attain a final volume of 2 ml. The final eluted volumes were collected and stored in sealed vials at proper temperatures until the time of HPLC analysis.

### 2.5. HPLC conditions

#### 2.5.1. AFB_1_ and OTA quantification by HPLC in spices and herbs samples

HPLC conditions were adopted from the instructions in the Aflaochraprep immunoaffinity columns manual supplied by R-Biopharm Rhone Ltd. (32). Reverse phase HPLC (Waters 2690^®^, Waters Corp., MA, USA) coupled with a fluorescence detector (Waters 2475^®^) and a HS C18 column (250 mm × 4.6 mm I.D., 5 μm particle diameter, Supelco Discovery^®^) fitted with a C18 guard column (Supelco Supelguard^®^, Sigma-Aldrich Co., MO, USA) at 25°C was used for analysis. The method for HPLC analysis was performed as described by the Aflaochra Prep IAC supplier R-Biopharm Rhone Ltd. Two mobile phases were used for analysis: solution A was composed of water: methanol (55:45 v:v), while solution B was composed of water: methanol (20:80 v:v). For both solutions, 119 mg potassium bromide and 350 μl of 4 M nitric acid were added for each 1 L. The mobile phases were prepared and filtrated on the same day of HPLC analysis, and they were passed at a flow rate of 0.8 ml/min. The sample injection volume was 100 μl and the wavelengths for excitation and emission at the beginning of the analysis were 365 and 442 nm, respectively, but after 17 min the excitation and emission were changed to 333 and 463 nm, respectively.

#### 2.5.2. AFB_1_ quantification by HPLC in nuts samples

HPLC conditions were adopted from the instructions in the Aflaprep immunoaffinity columns manual supplied by R-Biopharm Rhone Ltd. (33). Reverse phase HPLC (Waters 2690^®^, Waters Corp., MA, USA) coupled with a fluorescence detector (Waters 2475^®^) and a HS C18 column (250 mm × 4.6 mm I.D., 5 μm particle diameter, Supelco Discovery^®^) fitted with a C18 guard column (Supelco Supelguard^®^, Sigma-Aldrich Co., MO, USA) at 40°C temperature was used for analysis. The method for HPLC analysis was performed as described by the Aflaprep IAC supplier R-Biopharm Rhone Ltd. A mobile phase made of water: methanol (60:40 v:v) with 119 mg potassium bromide and 350 μl of 4 M nitric acid per liter was used for analysis. The mobile phase was prepared and filtrated on the same day of HPLC analysis, and it was passed at a flow rate of 1.0 ml/min. The sample injection volume was 100 μl and the wavelengths for excitation and emission were 362 and 425 nm, respectively.

### 2.6. Validation and quality assurance methods

A calibration curve was established using AFB_1_ and OTA standards at eight different concentrations ranging from 0 to 10 ppb. The limits of detection (LOD) and the limits of quantification (LOQ) were determined according to signal-to-noise ratios (S/N) of 3:1 and 6:1, respectively (34). The LOD and LOQ of AFB_1_ were 0.0054 and 0.018 μg/kg and for OTA 0.0030 and 0.014 μg/kg, respectively. Recovery analysis was performed were samples of spices, herbs, and nuts were spiked at three different levels of AFB_1_ and OTA. The average recovery for AFB_1_ and OTA were 82 and 90%, respectively. All tests, samplings, and operations in this study were conducted using the “Data Quality Assessment” checklist (35).

### 2.7. Population exposure to AFB_1_ and OTA

In order to assess the exposure of the Lebanese population to AFB_1_ and OTA, the food consumption data with regards to spices, herbs, and nuts was used to estimate the dietary exposure to AFB_1_ and OTA (35). In that context, a food frequency questionnaire (FFQ) was developed and verified to estimate the consumption rates of spices, herbs, and nuts in Lebanon. A random representative sample of 770 people was selected from all Lebanese governorates to participate in this study. Before starting the questionnaire, participants were clearly informed about the nature of the study, its objectives, methods, requirements, and guidelines. Additionally, an informed consent was obtained from every participant before filling the questionnaire. After obtaining the consumption data of every item, the exposure to AFB_1_ and OTA was evaluated accordingly:


$$\begin{array}{l} \text{ Average daily exposure to }{\text{AFB}}_{\text{1}}\text{/OTA from spices},\\ \text{herbs},\text{ or nuts }(\text{ng/person/day})\text{ = Average intake}\\ \;(\text{kg/person/day})\;\times \text{ Mean contamination}\\ \text{ with }{\text{AFB}}_{1}\text{/OTA }(\text{ng/kg}). \end{array}$$

The average daily exposure per person was then converted into average daily exposure to AFB_1_ and OTA per kg body weight per day based on the average weight of the Lebanese adult population found in this study which is 75.49 kg. This weight was used to calculate the average daily exposure to AFB_1_ and OTA per kg of body weight for adults of both genders.


$$\begin{array}{l} \text{ Daily exposure to }{\text{AFB}}_{\text{1}}\text{/OTA from spices},\text{ herbs},\text{and nuts }(\text{ng/kg body weight/day})\\ \text{= }\frac{\text{Average daily exposure to }{\text{AFB}}_{1}\text{/OTA from spices},\text{ herbs},\text{ and nuts }(\text{ng/person/day})}{\text{Average weight }(\text{kg})}. \end{array}$$

The average daily exposure to AFB_1_ and OTA from every item was summed up to get the total daily exposure (ng/kg body weight/day) from spices, herbs, and nuts in Lebanon.

The risk associated with exposure to AFB_1_ and OTA were analyzed according to the Joint FAO/WHO Expert Committee of Food Additives (JECFA) method. It is estimated that, for non-European countries, the ingestion of 1 ng per kg of body weight per day of aflatoxins would induce 0.083 liver cancer cases per year per 100,000 persons (36). So the liver cancer risk based on the total daily exposure to AFB_1_ (ng/kg body weight/day) from spices, herbs, and nuts was calculated as follows:


$$\text{Liver cancer risk = }\frac{\text{Exposure }(\text{ng/kg body weight/day})\;\times \text{ 0}.\text{083 cancer cases/100},\text{000 persons}}{\text{1 }(\text{ng/kg body weight/day})}.$$

As for OTA, JECFA established a provisional tolerable weekly intake (PTWI) of 100 ng/kg bw, so the risk resulting from OTA exposure was compared to this value (37).

### 2.8. Statistical analysis

The IBM^®^ SPSS^®^ software was used to compute means, range, standard deviations, and perform independent samples *t*-test to test significant difference on a *p-*value < 0.05.

## 3. Results

### 3.1. Spices

#### 3.1.1. AFB_1_

A summary of AFB_1_ results in spices is presented in Table 1. All of the 73 samples (100%) were found to contain AFB_1_ in the range of 0.13–18.35 μg/kg and at a mean of 0.97 μg/kg. Out of 73 samples only two nutmeg samples were contaminated above the MTL set by the EC.

**Table 1:** Incidence and occurrence of AFB_1_ in different kinds of spices in Lebanon.

**Spices**	**Samples analyzed** **(*n*)**	**Contamination level (**μ**g/kg)**		
		**Number (%) of samples exceeding MTL**	**Range of contamination**	**Mean** ±**SD**
Sweet pepper	13	**0**	0.14–0.96	0.23 ± 0.22
White pepper	10	**0**	0.15–3.11	0.51 ± 0.92
Black pepper	9	**0**	0.15–0.32	0.19 ± 0.06
Cinnamon	8	**0**	0.15–0.84	0.27 ± 0.23
Nutmeg	6	**2 (33.3%)**	1.65–18.35	7.28 ± 6.76
Turmeric	3	**0**	0.14–0.16	0.15 ± 0.01
Cumin	3	**0**	0.14–1.90	0.74 ± 1.00
Paprika	4	**0**	0.15–0.94	0.42 ± 0.37
Spices Mix	17	**0**	0.13–3.87	0.63 ± 0.93
Total	**73**	**2 (2.7%)**	0.13–18.35	0.97 ± 2.68

MTL for AFB_1_ in spices indicated in this table is 5 μg/kg.

Comparing different types of spices, all AFB_1_ mean contamination levels were found to be less than the MTL of 5 μg/kg, while only AFB_1_ mean contamination in nutmeg was found to be 7.28 μg/kg higher than the MTL.

#### 3.1.2. OTA

A summary of OTA contamination in spices is presented in Table 2. All of the 73 samples (100%) were found to contain OTA in the range of 0.12–452.46 μg/kg. The mean contamination of OTA in all spice samples was high at 38.8 μg/kg that exceeds the MTL set by the EC at 15 μg/kg. Out of 73 samples 22 samples (30.1%) were contaminated at levels above the MTL.

**Table 2:** Incidence and occurrence of OTA in different kinds of spices in Lebanon.

**Spices**	**Samples analyzed** **(*n*)**	**Contamination level (**μ**g/kg)**		
		**Number (%) of samples exceeding MTL**	**Range of contamination**	**Mean** ±**SD**
Sweet pepper	13	**1 (7.7%)**	0.47–44.22	15.08 ± 44.22
White pepper	10	**8 (80%)**	0.12–452.46	132.03 ± 141.79
Black pepper	9	**6 (66.7%)**	0.85–238.95	77.53 ± 89.08
Cinnamon	8	**0**	0.02–2.99	0.72 ± 0.95
Nutmeg	6	**4 (66.7%)**	1.39–236.26	68.05 ± 90.16
Turmeric	3	**0**	0.47–3.09	1.38 ± 1.50
Cumin	3	**1 (33.3%)**	0.12–31.99	11.11 ± 18.10
Paprika	4	**0**	0.13–3.62	1.36 ± 1.55
Spices mix	17	**2 (11.7%)**	0.49–79.45	9.33 ± 19.52
Total	**73**	**22 (30.1%)**	0.12–452.46	38.8 ± 80.5

MTL for OTA in spices indicated in this table is 15 μg/kg.

Comparing different types of spices, the highest mean contamination that exceeded the MTL of 15 μg/kg was recorded in white pepper (132.03 μg/kg), followed by black pepper (77.53 μg/kg), nutmeg (68.05 μg/kg), and sweet pepper (15.08 μg/kg). As for other types, the mean OTA level was below the MTL in cumin (11.11 μg/kg), spices mix (9.33 μg/kg), turmeric (1.38 μg/kg), paprika (1.36 μg/kg), and cinnamon (0.72 μg/kg).

### 3.2. Herbs

#### 3.2.1. AFB_1_

A summary of AFB_1_ results in herbs is presented in Table 3. Out of 54 samples, only 11 samples (20.4%) were found to contain AFB_1_ in the range of 0.72–4.87 μg/kg. The total mean of the 54 samples was low at 0.27 μg/kg.

**Table 3:** Incidence and occurrence of AFB_1_ in different kinds of herbs in Lebanon.

**Herbs**	**Samples analyzed** **(*n*)**	**Positive samples** ***n* (%)**	**Contamination level (**μ**g/kg)**	
			**Range of contamination**	**Mean** ±**SD**
Sumac	10	1 (10%)	BDL−0.84	0.08 ± 0.26
Thyme	10	3 (30%)	0.72–0.96	0.25 ± 0.41
Coriander	8	2 (25%)	0.85–2.16	0.38 ± 0.78
Dried mint	6	1 (16.7%)	BDL−0.89	0.15 ± 0.36
Oregano	6	1 (16.7%)	BDL−0.82	0.14 ± 0.33
Basil	3	0	–	–
Anise	8	2 (25%)	0.84–4.87	0.71 ± 1.71
Infused herbs	3	1 (33.3%)	BDL−0.82	0.27 ± 0.47
Total	**54**	11 (20.4%)	0.72–4.87	0.27 ± 0.76

#### 3.2.2. OTA

A summary of OTA results in herbs is presented in Table 4. Out of 54 samples, only 23 samples (42.6%) were found to contain OTA in the range of 0.02–23.82 μg/kg. The total mean of the 54 samples was low at 1.81 μg/kg.

**Table 4:** Incidence and occurrence of OTA in different kinds of herbs in Lebanon.

**Herbs**	**Samples analyzed** **(*n*)**	**Positive samples** ***n* (%)**	**Contamination level (**μ**g/kg)**	
			**Range of contamination**	**Mean** ±**SD**
Sumac	10	3 (30%)	0.02–1.24	0.24 ± 0.50
Thyme	10	3 (30%)	0.017–1.94	0.20 ± 0.61
Coriander	8	4 (50%)	0.017–10.98	1.50 ± 3.83
Dried mint	6	0	–	–
Oregano	6	2 (33.3%)	0.67–4.59	0.88 ± 1.84
Basil	3	2 (66.7%)	0.45–0.71	0.39 ± 0.36
Anise	8	8 (100%)	0.02–23.82	5.62 ± 8.30
Infused herbs	3	1 (33.3%)	BDL−20.18	10.04 ± 10.09
Total	**54**	23 (42.5%)	0.02–23.82	1.81 ± 4.78

### 3.3. Nuts

#### 3.3.1. AFB_1_

A summary of AFB_1_ results in nuts is presented in Table 5. Out of 71 samples, 70 were found to contain AFB_1_ at low levels in the range of 0.06–1.78 μg/kg and at a mean of 0.40 μg/kg. None of the samples were found to exceed the MTL set by the EC at 2 μg/kg for processed tree nuts and at 8 μg/kg for almonds and pistachios.

**Table 5:** Incidence and occurrence of AFB_1_ in different kinds of nuts in Lebanon.

**Product**	**Samples analyzed** **(*n*)**	**Positive samples** ***n* (%)**	**Contamination level (**μ**g/kg)**		
			**Number (%) of samples exceeding MTL**	**Range of contamination**	**Mean** ±**SD**
Mixed	9	9 (100%)	**0**	0.10–0.55	0.32 ± 0.16
Peanuts	20	19 (95%)	**0**	0.08–1.78	0.50 ± 0.46
Pistachios^a^	15	15 (100%)	**0**	0.18–0.84	0.41 ± 0.19
Cashews	6	6 (100%)	**0**	0.14–0.62	0.37 ± 0.18
Almonds^a^	11	11 (100%)	**0**	0.06–0.90	0.34 ± 0.28
Chickpeas	8	8 (100%)	**0**	0.06–0.50	0.34 ± 0.16
Others	2	2 (100%)	**0**	0.25–0.36	0.30 ± 0.08
Total	**71**	70 (98.6%)	**0**	0.06–1.78	0.40 ± 0.30

MTL for AFB_1_ in nuts indicated in this table is 2 μg/kg.

a The item specified has a MTL of 8 μg/kg.

### 3.4. Comparison between vacuum packed and un-packed samples

Table 6 shows the mean and range of contamination in vacuum packaged vs. unpackaged samples that were purchased from bulk, which is a common practice in Lebanon.

**Table 6:** Mean and range of contamination of AFB_1_ and OTA in vacuum packaged vs. unpackaged samples of spices, herbs, and nuts.

		**Spices**		**Herbs**		**Nuts**	
		**Packed** **(****51****)**	**Unpacked** **(****22****)**	**Packed** **(****34****)**	**Unpacked** **(****20****)**	**Packed** **(****28****)**	**Unpacked** **(****43****)**
AFB_1_	Mean ± SD (μg/kg)	0.86 ± 1.97	1.22 ± 3.91	0.26 ± 0.92	0.29 ± 0.41	0.34 ± 0.20	0.44 ± 0.34
	Range (μg/kg)	0.13–12.90	0.14–18.40	0.07–4.87	0.84–0.96	0.08–0.90	0.06–1.78
	*p-*value (packed vs. unpacked)	0.59^*^		0.11^*^		0.143^*^	
OTA	Mean ± SD (μg/kg)	40.8 ± 90.0	34.0 ± 80.50	2.23 ± 5.52	1.10 ± 3.18		
	Range (μg/kg)	0.12–452	0.02–162	0.02–23.8	0.02–10.8		
	*p-*value (packed vs. unpacked)	0.741^*^		0.881^*^			

* All p-values are >0.05 indicating no significant difference between packed and unpacked sample means.

For AFB_1_, it was evident that in spices, herbs, and nuts the mean contamination levels found in unpackaged samples were higher than packaged ones, however, those differences were not statistically significant (*p* > 0.05).

As for OTA, the mean contamination levels found in packaged samples of spices and herbs were higher than unpackaged ones, however, those differences were also not statistically significant (*p* > 0.05).

### 3.5. Exposure level

The consumption levels of spices, herbs, and nuts, the average contamination of each item, and the exposure to AFB_1_ and OTA are presented in Table 7.

**Table 7:** Average consumption of spices, herbs, and nuts, average contamination of AFB_1_ and OTA, and exposure level to AFB_1_ and OTA.

**Type**	**Consumption (g/person/day)**	**AFB_1_ contamination (ng/kg)**	**Exposure to AFB_1_ (ng/person/day)**	**OTA contamination (ng/kg)**	**Exposure to OTA (ng/person/day)**
Spices	1.33	967	1.29	38,765	51.6
Herbs	2.56	164	0.42	1,205	3.08
Nuts	33.6	398	13.4	0	0
**Total**			**15.1**		**54.6**

The total exposure to AFB_1_ was shown to be 15.1 ng/person/day so accordingly, the daily exposure was calculated in our study to be 0.2 ng/kg bw/day. Therefore, this exposure would be associated with 0.017 additional liver cancer cases per 100,000 persons per year.

On the other hand, the total exposure to OTA was shown to be 54.6 ng/person/day so accordingly, the daily exposure was calculated in our study to be 0.72 ng/kg bw/day, and therefore the weekly exposure is 5.04 ng/kg bw much less than the PTWI of 100 ng/kg bw set by JECFA.

## 4. Discussion

In this study, AFB_1_ was found in 100% and 20.4% of spices and herbs, respectively, with two nutmeg samples (2.7% of total spices samples) exceeding MTL of 5 μg/kg in spices. The mean concentration of AFB_1_ in spices was reported to be 0.97 μg/kg higher than that in herbs of 0.27 μg/kg. All types of spices had mean concentrations at levels below the limit of 5 μg/kg except nutmeg samples in which the AFB_1_ mean of 7.28 μg/kg exceeded the limit.

**Figure 1:**
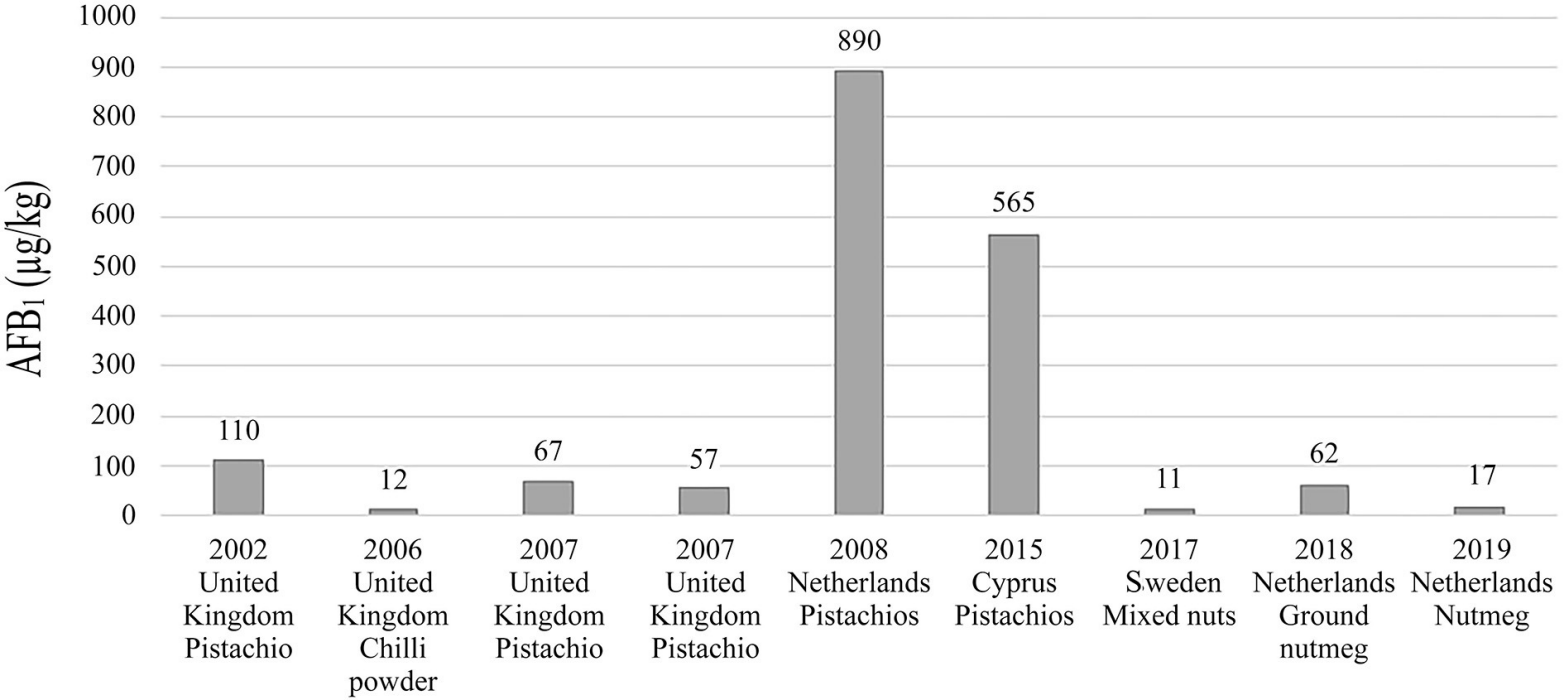
Examples of reported border rejections on RASFF portal of Lebanese imports to the European Union due to high levels of AFB_1_ in spices, herbs, and nuts.

While, In Lebanon, a previous study done, reported the contamination of 15.9 and 8% of spices and herbs samples with AFB_1_, respectively, with 14 and 8% of samples exceeding MTL set by the EC (25). As mentioned previously, AFB_1_ contamination in Lebanese products of spices and nuts was also reported on the RASFF portal (28). Figure 1 shows examples of rejected imports and the concentration of AFB_1_ in each.

Several studies around the world reported contamination with AFB_1_ in spices, for example, studies done in Iran and Hungary showed that 13.3 and 16.5% of samples, respectively, exceeded MTL, which was much higher than that reported in this study (2.7%) (38, 39). In Morocco all tested samples were found contaminated, which is similar to our result (40). However, data from another countries in the world reported less contamination such as in Turkey, Italy, Spain, Portugal, and Brazil where AFB_1_ was found in 96, 46, 90, 43, and 61% of spices samples, respectively (41–45). As for herbs, few studies were performed worldwide; for example, AFB_1_ was found in 29% and 93.3% of herb samples in Egypt and India, respectively (46, 47).

In nuts, 98.6% of samples contained AFB_1_ at levels below MTL of 2 μg/kg and a low mean concentration of 0.40 μg/kg. Previously, Raad et al. reported contamination of nuts with AFB_1_ in Lebanon in which a composite group made up of nuts, seeds, olives, and dried dates had AFB_1_ at a mean concentration of 0.1 μg/kg (27). While Soubra et al. found that contamination with AFB_1_ in nuts ranged from 0.5 to 8.0 μg/kg and the mean was 1.0 μg/kg higher than the level reported in this study (26). The variation of results between this study and other ones might be due to differences in sampling periods, methods, preparation, and analysis. Moreover, high levels of AFB_1_ have been also reported in Lebanese nut imports to the European Union (Figure 1) (28). Around other parts of the world contamination studies reported, as well, the occurrence of AFB_1_ in nuts. For example, similar levels as this study were found in Iran and Brazil, where 96.5% of samples were found contaminated in both countries (48, 49). While in Libya and Pakistan lower contamination levels were found with 31.1% and 45% of samples contaminated with AFB_1_ (50, 51). However, in Pakistan and Brazil AFB_1_ contamination exceeded MTL in 39.5% and 2.8% of samples (48, 50), respectively, while in this study no sample was reported to contain AFB_1_ at levels above this limit.

Comparing the packaged and unpackaged samples, AFB_1_ mean concentration was higher in unpackaged samples for spices, herbs, and nuts, but this difference was not statistically significant.

On the other hand, OTA was prevalent in 100% of spices with 30.1% exceeding MTL (one sweet pepper, eight white pepper, six black pepper, four nutmeg, one cumin, and two spices mix samples). The mean concentration of OTA in spices was high at 38.8 μg/kg. The highest mean OTA concentration was reported in white pepper (132.03 μg/kg), followed by black pepper (77.53 μg/kg), nutmeg (68.05 μg/kg), sweet pepper (15.08 μg/kg), cumin (11.11 μg/kg), spices mix (9.33 μg/kg), turmeric (1.38 μg/kg), paprika (1.36 μg/kg), and cinnamon (0.72 μg/kg).

OTA was found in 44.4% of herb samples at a low mean concentration of 1.81 μg/kg, however, two kinds of herbs, namely, infused herbs and anise, had a higher mean concentration of OTA than other kinds at 10.04 and 5.62 μg/kg, respectively.

Previously in Lebanon, Darra et al., reported that 29.8% of spices samples contained OTA with 3% of samples exceeding MTL, while 10.5% of herbs were contaminated with no sample exceeding MTL. Furthermore, spices were contaminated with OTA at a range of 0.7–33.9 μg/kg (25), lower than the contamination range found in this study that was between 0.02 and 452 μg/kg. As for herbs, the range of contamination reported by Darra et al. (25) of 0.7–9.8 μg/kg was lower as well than that reported in this study between 0.02 and 23.8 μg/kg. The variation between this study and the previous one is related to differences in sampling period, method, and analysis.

Other studies done around the world reported contamination with OTA in spices, for example, in Hungary, 71.4% of samples were contaminated with 17.6% exceeding MTL (38). In other countries studies reported less contamination as well, such as in Tunisia, Spain, Korea, Malaysia, and Brazil where OTA was found in 70, 98, 22, 82, and 86 of spices samples, respectively (45, 52–55). As for herbs, 42.6% of samples were found contaminated with OTA in India, similar to this study with 44.4% positive samples, while lower levels were reported in Croatia where 14.3% of samples were contaminated (56, 57).

Since Lebanon imports spices, the high occurrence levels of OTA might indicate local contamination due to improper storage practices such as inappropriate temperature and humidity conditions in factories and/or in the market. This may allow for further contamination with OTA while in storage especially in traditional local shops where batches of spices and herbs are kept mostly in unsealed containers at room temperatures.

Exposure to AFB_1_ and OTA, from the tested samples, was also reported in this study. The exposure to AFB_1_ from spices, herbs, and nuts was found to be 0.2 ng/kg bw/d which would be associated with 0.017 additional liver cancer cases per 100,000 persons per year. On the other hand, the total exposure to OTA was shown to be 54.6 ng/person/day so accordingly, the daily exposure was calculated in our study to be 0.72 ng/kg bw/day, and therefore the weekly exposure is 5.04 ng/kg bw much less than the PTWI of 100 ng/kg bw set by JECFA indicating low risk of renal damage from spices and herbs consumption.

Compared to findings from other studies, the exposure to AFB_1_ from spices was found to be 0.017 ng/kg bw/d which is much lower than the value of 1.55 ng/kg bw/d reported in Lebanon by Al Ayoubi et al. (58). On the other hand, OTA exposure form spices was reported in this study to be 0.68 ng/kg bw/d at six times fold greater than the one determined by Al Ayoubi et al. (58) and reported to be 0.11 ng/kg bw/d. This variance in findings between the two studies could be due to the difference in tested sample types, methods of testing, and analysis.

## 5. Implications and control strategies

In this study AFB_1_ was reported at low levels in spices, herbs, and nuts in which the mean concentration was 1.0, 0.3, and 0.4 μg/kg, respectively. However, OTA was reported at high levels in spices at a mean concentration of 38.8 μg/kg, unlike herbs that had OTA at a mean concentration of 1.8 μg/kg. Therefore, the consumption of these commodities in Lebanon, especially spices, could lead to an increase in health risks associated with AFB_1_ and OTA knowing that these food products are extensively consumed by the Lebanese population. The exposure and effect of AFB_1_ and OTA are studied separately in this study, but nonetheless, their co-occurrence could lead to synergistic effects on health, therefore, causing additional health risks on Lebanese consumers. Added to that, other food products than spices, herbs, and nuts could contain AFB_1_ and OTA such as wheat, wheat-derivatives, dried fruits, coffee, wine, beer… and could add to the exposure to these toxins, therefore, increasing the health risks correlated with them.

The reported occurrence of AFB_1_ and OTA may be directly related to poor storage conditions that promote fungal invasion and mycotoxin production. Therefore, regular inspections are required especially on warehouses, factories, supermarkets, roasteries, and local shops to enforce the application of good storage practices.

Finally, continuous monitoring of AFB_1_ and OTA in spices, herbs, and nuts in Lebanon should be implemented and studies should be done routinely to ensure their safety.

## 6. Conclusion

AFB_1_ and OTA contamination in food can present a risk to public health and this study showed contamination of spices, herbs, and nuts in Lebanon at different levels. Therefore, assuring the safety of those food items consumed at regular basis by Lebanese population is essential to prevent health problems related specifically to chronic exposure to AFB_1_ and OTA. Hence, continuous monitoring and inspections are recommended.

## Data availability statement

The raw data supporting the conclusions of this article will be made available by the authors, without undue reservation.

## Author contributions

RD contributed to application, statistical analysis, and writing of the study. AK contributed to the planning, supervision, project administration, reviewing, writing, and approving of final version. RM and AI contributed to supervision, reviewing, and approving of final version. MH, KB, KJ, and LK contributed to reviewing and approving of final version. All authors contributed to the article and approved the submitted version.
